# Naloxone should remain the appropriate antidote to treat opioid overdose

**DOI:** 10.1186/s13054-020-2835-5

**Published:** 2020-04-28

**Authors:** Bruno Mégarbane, Lucie Chevillard, Dominique Vodovar

**Affiliations:** 1 Department of Medical and Toxicological Critical Care, Lariboisière-Fernand Widal Hospital, Federation of Toxicology APHP, Paris University, 2 Rue Ambroise Paré, 75010 Paris, France; 2 INSERM UMRS-1144, Paris University, Paris, France; 3 Poison Control Center of Paris, Lariboisière-Fernand Widal Hospital, Federation of Toxicology APHP, Paris University, Paris, France

To the Editor,

In a randomized trial, Zamani et al. assessed the greater effectiveness and safety of buprenorphine versus naloxone to reverse methadone overdose-related respiratory depression [1]. We congratulate the authors. However, several issues may limit the accuracy of their observations and recommendation to unselectively treat opioid overdoses with buprenorphine.

Usually, opioid overdose is identified in patients presenting stupor or coma (i.e., Glasgow coma score [GCS] ≤ 12), respiratory rate [RR] ≤ 12/min and miosis) [2]. Zamani’s patients were mildly poisoned with mean/median (not clear) RR of 12–15/min and GCS of 13–15. Therefore, the elevated naloxone-induced withdrawal rate clearly resulted from excessive dosage and/or inadequate titration aiming to obtain “complete arousal to normal consciousness” in mildly overdosed opioid-dependent patients. Opioid-tolerant patients frequently respond to low naloxone doses, sufficient to restore breathing without provoking withdrawal [2]. Clinicians incorrectly assume that the naloxone dose required to restore respiration correlates with severity. Naloxone should be injected at 0.04 mg bolus and titrated/2 min upward to effect (i.e., RR ≥ 15/min).

Naloxone-attributed complications were surprisingly frequent, not reflecting our practice, especially since patients with cardiovascular morbidities, co-ingestions, aspiration, and need for immediate intubation were excluded. ARDS onset in 15% of naloxone-treated patients without prior aspiration is unusual. Naloxone has been mistakenly implicated as a cause of pulmonary edema [2]. Using optimal regimens allows the restoration of oxygenation and prevents the postulated sympathetic surge that triggers pulmonary edema after apnea reversal.

In Fig. 2, 13 apnea episodes were reported in the naloxone group, mostly reversed by naloxone boluses. Management of the 11 apnea episodes in the buprenorphine groups was not provided since only five patients were intubated. Did the patients receive buprenorphine boluses or continuous infusion?

Intravenous buprenorphine should be used cautiously in opioid users who, unlike the Iranian experience, mostly co-abuse benzodiazepines and/or ethanol [3]. Drug-drug interactions and intravenous buprenorphine administration are the two well-recognized circumstances that impair buprenorphine-related ceiling effects, worsening ventilation consequently, as demonstrated experimentally [4, 5]. Because naloxone is lacking agonist activity on the mu-opioid receptor, no such risk exists. Additionally, in non-methadone abusers, buprenorphine may compete with the existing opioid on the mu-opioid receptor and, due to higher binding affinity, produce late withdrawal if the opioid exhibits shorter elimination half-life.

Therefore, unless convincing data are produced, naloxone should remain the legitimate antidote to reverse opioid toxicity. Concerns that naloxone may harm opioid-dependent patients are unfounded [2]. With adequate titration, signs of naloxone-induced abstinence are unpleasant but never life-threatening.

## Authors’ response

Nasim Zamani^1,2,3^, Nicholas A. Buckley^4^ and Hossein Hassanian-Moghaddam^1,2^

^1^Social Determinant of Health Research Center, Shahid Beheshti University of Medical Sciences, Tehran, Iran

^2^Department of Clinical Toxicology, Loghman Hakim Hospital, Shahid Beheshti University of Medical Sciences, South Karegar Street, Tehran, Iran

^3^Toxicological Research Center, Shahid Beheshti University of Medical Sciences, Tehran, Iran

^4^Pharmacology, Faculty of Medicine and Health, University of Sydney, Sydney, Australia

To the Editor,

The comments mentioned are a reflection of the opinions of three clinicians working in a Paris ICU [1]. That is a setting typically many hours post-emergency resuscitation, and many of the complications with naloxone may fall outside their remit. Iran has a 100-fold higher rate of fatal/non-fatal methadone overdose than France. The reported rate of methadone-related hospitalization was just 5.4 per 1,000,000 French inhabitants in 2017 [6]. Around 31 per 1,000,000 people died in Tehran due to methadone overdose in 2015, and this would be just a fraction of the total overdoses [1, 7].

It is not the lack of experience that creates problems with managing respiratory depression without precipitating in withdrawal using titrated naloxone [1]. Methadone overdose is inherently difficult to manage with naloxone; it has a long and variable half-life with a resulting wide range of concentrations [7]. As seen in our article, the range of doses and durations of naloxone required both vary more than ten-fold. This contrasted with the simple (but more effective) buprenorphine dosing.

There is published evidence on the complications of naloxone particularly in higher doses [8]. A US study reported pulmonary complications in 26.5% of 1831 patients. Those receiving doses > 4.4 mg had a 46% complication rate [7]. Due to the long half-life of methadone, most of our patients required such higher doses of naloxone to overcome opioid effects.

We reported GCS at the time of randomization, but this often reflected a preceding dose of naloxone in the prehospital setting. Evidence for severe toxicity requiring effective reversal is seen in rates of apnea (22%), intubation (30%), prolonged sedation (33%), and ARDS (15%). ARDS may be secondary to further apnea and aspiration or even as a result of higher doses of naloxone infusion [8]. Further, 15% of those treated with naloxone died. In terms of apnea reversal, in each group, apnea was reversed by naloxone or buprenorphine after hospitalization accordingly [1].

Megarbane et al. also raise two theoretical points based on rat studies. We believe the theory also indicates concerns about precipitating withdrawal and interacting drugs and alcohol apply to an even greater extent for naloxone. In contrast, using sublingual buprenorphine in the ED is beneficial in starting the process of buprenorphine maintenance therapy and thus in harm reduction and reducing further overdoses and deaths [9]. Further evidence from other settings are definitely needed to withdraw more robust results.

Yours truly,

Nasim Zamani, Nick A Buckley, and Hossein Hassanian-Moghaddam.

## Data Availability

Not applicable
